# The Fragile X Mental Retardation Protein Regulates Striatal Medium Spiny Neuron Synapse Density and Dendritic Spine Morphology

**DOI:** 10.3389/fnmol.2020.00161

**Published:** 2020-09-10

**Authors:** Jessica L. Huebschman, Kitzia S. Corona, Yuhong Guo, Laura N. Smith

**Affiliations:** ^1^Department of Neuroscience and Experimental Therapeutics, Texas A&M University Health Science Center, Bryan, TX, United States; ^2^Texas A&M Institute for Neuroscience, Texas A&M University, College Station, TX, United States

**Keywords:** striatum, dendritic spine, RNA-binding proteins, FXS, synapse structure, morphology

## Abstract

The fragile X mental retardation protein (FMRP), an RNA-binding protein that mediates the transport, stability, and translation of hundreds of brain RNAs, is critically involved in regulating synaptic function. Loss of FMRP, as in fragile X syndrome (FXS), is a leading monogenic cause of autism and results in altered structural and functional synaptic plasticity, widely described in the hippocampus and cortex. Though FXS is associated with hyperactivity, impaired social interaction, and the development of repetitive or stereotyped behaviors, all of which are influenced by striatal activity, few studies have investigated the function of FMRP here. Utilizing a cortical-striatal co-culture model, we find that striatal medium spiny neurons (MSNs) lacking FMRP fail to make normal increases in PSD95 expression over a short time period and have significant deficits in dendritic spine density and colocalized synaptic puncta at the later measured time point compared to wildtype (WT) MSNs. Acute expression of wtFMRP plasmid in *Fmr1* KO co-cultures results in contrasting outcomes for these measures on MSNs at the more mature time point, reducing spine density across multiple spine types but making no significant changes in colocalized puncta. FMRP’s KH2 and RGG RNA-binding domains are required for normal elimination of PSD95, and interruption of these domains slightly favors elimination of immature spine types. Further, KH2 is required for normal levels of colocalized puncta. Our data are largely consistent with a basal role for FMRP and its RNA-binding domains in striatal synapse stabilization on developing MSNs, and in light of previous findings, suggest distinct regional and/or cell type-specific roles for FMRP in regulating synapse structure.

## Introduction

The fragile X mental retardation protein (FMRP) is an RNA-binding protein encoded by the *Fmr1* gene, which regulates the transport, stability, and translation of hundreds of brain RNAs, many of which are critically involved in synaptic function. FMRP contains several RNA-binding motifs, including three K-homology (KH0, KH1, and KH2) domains and a C-terminal arginine-glycine-glycine (RGG) box, which associate with “kissing complex” and “G-quadruplex” RNA structures, respectively (Schaeffer et al., 2001; Darnell et al., 2005a; Myrick et al., 2015). Loss of FMRP, as observed in fragile X syndrome (FXS), is a leading monogenic cause of autism and intellectual disability, and is characterized by altered structural and functional synaptic plasticity throughout the brain (Irwin et al., 2001; Nimchinsky et al., 2001; Huber et al., 2002; Antar et al., 2006; Grossman et al., 2006; Pfeiffer et al., 2010; Bagni et al., 2012; Smith et al., 2014; Neuhofer et al., 2015). Of note, a single point mutation in FMRP’s KH2 domain (I304N) reliably abolishes its interactions with “kissing complex” RNAs and polyribosomes *in vitro* (Darnell et al., 2005a), though no kissing complex motifs have yet been identified in FMRP targets, including those in a high confidence dataset (Darnell et al., 2011; Ascano et al., 2012; Anderson et al., 2016; Maurin et al., 2018). However, the I304N mutation does result in a particularly severe form of FXS (De Boulle et al., 1993; Feng et al., 1997), and truncations and point mutations in the RGG domain have been identified in individuals exhibiting FXS symptoms (Handt et al., 2014; Okray et al., 2015; Suhl and Warren, 2015), implicating both domains in the disease pathology.

Abnormalities in synapse density and dendritic spine morphology have been widely reported in human patients and animal models of FXS, with most studies indicating an increase in synapse and spine density, particularly of immature or transient spine types, throughout the cortex and hippocampus (Antar et al., 2006; Grossman et al., 2006; Pfeiffer and Huber, 2007; He and Portera-Cailliau, 2013). FMRP also regulates mGluR5-dependent hippocampal long-term depression (Huber et al., 2002) and changes in cortical GluR1 surface expression (Wang et al., 2008). In cultured *Fmr1* knockout (KO) hippocampal cells, acute expression of wildtype (WT) FMRP reduces synapse number in a manner dependent on its KH2, but not its RGG, RNA-binding domain (Pfeiffer and Huber, 2007), while FMRP having both the KH2 and RGG domains intact is required for activity-dependent synapse elimination by the myocyte enhancer factor 2 (MEF2) transcription factor (Pfeiffer et al., 2010), differentially implicating these domains in various forms of synaptic plasticity. FMRP also regulates numerous presynaptic activities, including translation-independent ion channel trafficking and stabilization, neurotransmitter release, and axon growth cone dynamics (Antar et al., 2006; Centonze et al., 2008; Deng et al., 2013; Ferron et al., 2020). Interestingly, while FMRP regulates localization of presynaptic voltage-gated calcium channels independently of new protein synthesis, mutations in the RGG RNA binding domain are sufficient to impair the protein-protein interactions necessary for this function (Ferron et al., 2020), highlighting a broader role for these domains in regulating synaptic plasticity.

Few studies have examined FMRP’s function outside of the cortex and hippocampus. However, FXS is associated with hyperactivity, impaired social interaction, and the development of repetitive, or stereotyped behaviors, all of which are influenced by striatal activity, suggesting that FMRP may regulate synaptic function in this region, as well (Langen et al., 2011; Bagni et al., 2012; Báez-Mendoza and Schultz, 2013; Yael et al., 2019). Inhibitory transmission is enhanced in the striatum of *Fmr1* KO mice, despite a significant decrease in the number of GABAergic synapses (Centonze et al., 2008), but little is known about FMRP’s striatal role in regulating plasticity. In the ventral striatum, lack of FMRP has been associated with deficits in presynaptic function and decreased stubby-type dendritic spine density (nucleus accumbens, shell region; Smith et al., 2014), a sharp contrast with the increases in spine density reported in cortex and hippocampus. While these initial studies suggest it may have a unique striatal function, the full extent of FMRP’s regulation of synapses throughout this region remains largely unknown. Here, we sought to establish the role of FMRP in regulating striatal excitatory synapse number and dendritic spine morphology, and determine whether this function is dependent upon the KH2 or RGG RNA-binding motifs.

## Materials and Methods

### Animals

Trio or pairwise breeding was conducted under standard laboratory conditions, in ventilated cages with a 12-h light/dark cycle (on at 06:00) and *ad libitum* access to standard mouse chow and water. Littermate embryos on a C57BL/6N background, either WT (male) and *Fmr1* KO (male and female) or *Fmr1* KO only (male and female), were generated with *Fmr1*^−/y^ and *Fmr1*^−/+^ or *Fmr1*^−/y^ and *Fmr1*^−/^ breeders, respectively. All procedures were conducted in compliance with the Texas A&M University Institutional Animal Care and Use Committee (Protocol #: 2017-0234).

### Primary Cortical-Striatal Co-culture

Dissociated cortical-striatal co-cultures were prepared on embryonic day 16 (ED16) using previously described protocols (Penrod et al., 2011). Briefly, pregnant mice were euthanized by CO_2_ asphyxiation, and embryos were removed and rapidly genotyped by PCR. Cortical tissue (roughly corresponding to the prefrontal cortex) and the combined medial and lateral ganglionic eminences were collected by region and genotype into separate 15 ml conical tubes containing fresh Ca^2+^/Mg^2+^-free Hank’s Balance Salt Solution with 10 mM HEPES (CMF-HBSS). After tissue had settled to the bottom of the tubes, CMF-HBSS was replaced with 0.25% trypsin digestion solution (10× Trypsin-EDTA, Sigma–Aldrich T4174). Tissue was incubated in digestion solution for 30 min at 37°C, after which cells were centrifuged at 1,000× *g* for 5 min and digestion solution was replaced with neuronal plating media (10 mM HEPES, 10 mM sodium pyruvate, 0.5 mM glutamine, 12.5 μM glutamate, 10% Newborn Calf Serum, 0.6% glucose in Earl’s Minimal Essential Media). Following trituration, dissociated cells were counted using trypan blue and a hemocytometer. Cells were plated on coverslips coated with poly-D-lysine (PDL; Fisher ICN10269410) and laminin (Thermo Fisher 23017015) at a total density of 2 × 10^5^ cells/35 mm dish (two parts striatal to three parts cortical). After 1 h, plating media was replaced with neuronal growth media [Neurobasal, Thermo Fisher 21103049; 50× B27, Thermo Fisher 17504-044; 0.5 mM L-glutamine (Q), Sigma G7513]. Cultures were kept at 37°C/5% CO_2_ and half of the media per plate was replaced every 3–4 days with new growth media. Cultures were designated for either synaptic puncta or dendritic spine analysis; for each measurement, two to three replicates (separate cultures/litters) were used per group. In relevant studies, calcium phosphate transfection was performed at day *in vitro* (DIV) eight to introduce green fluorescent protein (GFP) alone, or various forms of enhanced (E)GFP-tagged FMRP. The WT (wtFMRP-EGFP), arginine/glycine-rich box (RGG) deletion (lacking amino acids RRGDGRRRGGGGRGQGGRGRGGGFKGN; ΔRGG-FMRP-EGFP), and I304N point mutant (KH2 domain; I304N-FMRP-EGFP) forms of FMRP-EGFP were under control of the endogenous human *Fmr1* promoter, as described (Darnell et al., 2005b; Pfeiffer and Huber, 2007). For spine analysis, cells were transfected with mCherry, either alone or in addition to the above constructs, at the same time point. At designated time points (DIV 10 or 14), cells were fixed in 4% paraformaldehyde (PFA)/4% sucrose (15 min) at room temperature, washed in 1× PBS (three times), and stored at 4°C protected from light until staining.

### Immunocytochemistry and Fluorescent Microscopy

For synaptic puncta experiments, fixed cells were blocked in 10% goat serum (30 min) and permeabilized in 0.2% Triton-X (10 min) at room temperature. A second blocking step (10 min) was used, as recommended for the preservation of fine cell structures (Penrod et al., 2011). Cells were incubated overnight (shaking, 4°C) in primary antibody diluted in PBS with 1% goat serum (synapsin Ia and Ib, Millipore Sigma AB1543, 1:500; PSD95 Millipore Sigma MABN 68, 1:200). Afterwards, cells were rinsed with PBS and incubated (1 h) at room temperature in goat anti-rabbit Alexa-Fluor 647, goat anti-mouse Alexa-Fluor 488 (untransfected), or goat anti-mouse Alexa-Fluor 594 (transfected) secondary antibodies (Thermo Fisher, 1:1,000) diluted in PBS with 1% goat serum and 0.2% Triton-X. Following final rinses in PBS, coverslips were mounted on microscope slides with Vectashield antifade mounting medium (Vector Labs H-1000) for imaging.

Fluorescence was detected using an Olympus FV1000 confocal microscope with a 60× (puncta) or 100× (spines) oil immersion lens. Intact medium spiny neurons (MSNs) were identified for analysis by their soma size (10–20 μm) and dendritic arborization, as indicated by expression of PSD95 (puncta, untransfected cells), GFP (puncta, transfected cells), or mCherry (spines). For synaptic puncta experiments, *z*-stacks encompassing the entirety of the cell soma and visible processes were collected using a step size of 0.50 μm. For dendritic spine experiments, a *z*-stack (step size 0.45 μm) of mCherry fluorescence from an isolated region of a single secondary or higher order dendritic branch (≥20 μm) from each cell was collected for analysis. To minimize crossover of GFP fluorescence emission, GFP+ cells were first identified *via* epifluorescence and then, using the 543 nm laser, mCherry positivity was confirmed and a *z*-stack of the dendritic branch was collected. When collecting a multi-channel image that included both GFP and mCherry signals, mCherry was first collected alone for reconstruction before proceeding to multi-channel imaging, minimizing photobleaching and ensuring that images collected for spine analyses were captured with only the 543 nm laser active.

### Synaptic Puncta Analysis

Maximum intensity projection images were generated in Fiji (Schindelin et al., 2012) from acquired *z*-stacks using the Extended Depth of Field plugin (Aguet et al., 2008), and used to generate cell reconstructions with the NeuronJ plugin (Meijering et al., 2004). Reconstructions of MSNs were built from either the PSD95 (basal experiments) or GFP (transfection experiments) projection images. Points of fluorescent intensity above a set threshold for PSD95 and synapsin I, as well as points of colocalization, defined as overlap between PSD95 and synapsin I signals, were quantified along dendritic tracings in a radius of approximately 70 μm in all directions from the soma of interest using the SynapCountJ2 plugin (Mata et al., 2016). Dendritic diameter was set to 20 pixels, or approximately 3.12 μm, and only points of fluorescent intensity above threshold within this dendritic area were included in analysis. Thresholds were defined for each cell individually as a set number of standard deviations (*untransfected cells*: 3 or 4; *transfected cells*: 2 or 3; for PSD95 and synapsin I, respectively) above the average fluorescent signal from that cell’s maximum intensity projection image, not varying within experiment.

### Dendritic Spine Analysis

MSN dendritic branches and spines were reconstructed from *z*-stacks using the semi-automated Filament Tracer tool in Imaris (Bitplane, Oxford Instruments). Morphological characteristics, including spine head and neck diameter, branching, and length were used to classify dendritic spines based on previously described criteria (Harris et al., 1992; Jedynak et al., 2007). Briefly, spines having head diameters ≥0.55 μm, which also exceeded the diameter of the spine neck, were considered mushroom type. Spines with a spine head <0.55 μm but still greater than the spine neck diameter were classified as thin type. When head diameter equaled or was less than spine neck diameter, length determined categorization as either filopodia (>1.0 μm) or stubby type (≤1.0 μm). Spines with single or multiple branch points were categorized as branched or thorny, respectively, regardless of other measurements. Spine density was calculated for each cell as the number of spines (total or by type) over the length of the reconstructed dendritic branch.

### Statistical Analysis

All statistical analysis and results are listed in Supplementary Table S1. Synaptic puncta and total dendritic spine density were analyzed with one- (plasmid) or two-way (genotype × time point) between-subjects analysis of variance (ANOVA). Densities of spines by type were analyzed using two-way (genotype × spine type or plasmid × spine type) mixed ANOVAs, where spine type was a within-subjects variable. Significant interactions were followed by additional ANOVAs (one-way), paired *t*-tests, and/or Bonferroni *post hoc* analyses, as appropriate, to evaluate simple main effects (SMEs). When Mauchly’s test of sphericity was significant, either Greenhouse–Geisser (G–G; when ε < 0.75) or Huynh–Feldt (H–F; when ε > 0.75) corrections were used. All statistics were performed using GraphPad Prism or SPSS software. Significance was set at α = 0.05.

## Results

### FMRP Mediates Striatal Excitatory Synapse Number

**Figure 1:**
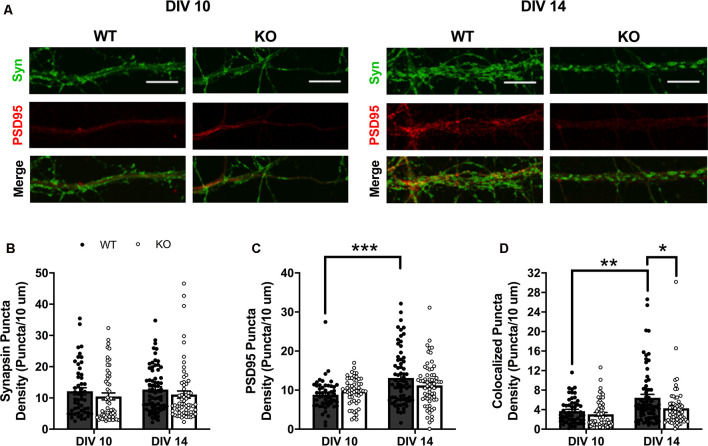
Expression of presynaptic (synapsin I; green) and postsynaptic (PSD95; red) markers in wildtype (WT) and *Fmr1* knockout (KO) medium spiny neurons (MSNs) at day *in vitro* (DIV) 10 and 14 (**A**; scale bars are 10 μm). Synapsin I expression did not vary between genotypes or across the two time-points (**B**; two-way analysis of variance, ANOVA). WT cells have significantly increased density of PSD95 at DIV 14 compared to DIV 10, but KO cells show no change in expression level between the two time points (**C**; two-way ANOVA). Only WT cells increase the density of colocalized pre- and postsynaptic markers from DIV 10 to 14, with KO cells showing a significant deficit at DIV 14 compared to WT counterparts (**D**; two-way ANOVA). Significant Bonferroni comparisons are indicated (*p < 0.05, **p < 0.01, ***p < 0.001); data shown are mean ± SEM.

To determine FMRP’s role in regulating excitatory synapse number, we compared expression of presynaptic (synapsin I) and postsynaptic (PSD95) markers, quantified as distinct puncta above a set threshold (see “Materials and Methods” section), along dendritic branches of cultured WT and *Fmr1* KO MSNs at DIV 10 and 14 (Figure 1A). Synapsin Ia is fairly stably expressed, at least in cultured embryonic hippocampal cells, over the time points represented here (Ferreira et al., 2000), and with little exception, synapsin I is expressed in all neurons (presynaptic), and appearance is tightly linked to synaptic ontogenesis (De Camilli et al., 1983). There is additional evidence in other culture systems that pre- and postsynaptic proteins are stably present at or before DIV 10, largely preceding, but ultimately associated with, the development of spines or synapses (Ahmari et al., 2000; Prange and Murphy, 2001). We observed no significant main effects or interactions of genotype or day on expression of the presynaptic marker, synapsin I (Figure 1B). For expression of the postsynaptic marker, PSD95, two-way ANOVA showed a significant interaction between day and genotype (*F*_(1,236)_ = 4.13, *p* < 0.05). Follow-up Bonferroni analysis of this interaction revealed a significant SME of day for WT cells (*p* < 0.001), with density of PSD95 puncta being significantly higher on MSNs at DIV 14 (Figure 1C). We next quantified density of colocalized synapsin I and PSD95 puncta staining, as a measure of structural excitatory synapses. Two-way ANOVA of colocalized puncta revealed significant main effects of day (*F*_(1,236)_ = 13.13, *p* < 0.001) and genotype (*F*_(1,236)_ = 6.639, *p* < 0.05; Figure 1D). SMEs were observed for day within the WT group (*p* < 0.01), with DIV 14 greater than DIV 10, and for genotype within the DIV 14 time point (*p* < 0.05), where WT had significantly more colocalized puncta than *Fmr1* KO cells.

### Striatal *Fmr1* KO MSNs Have Reduced Dendritic Spine Density

**Figure 2:**
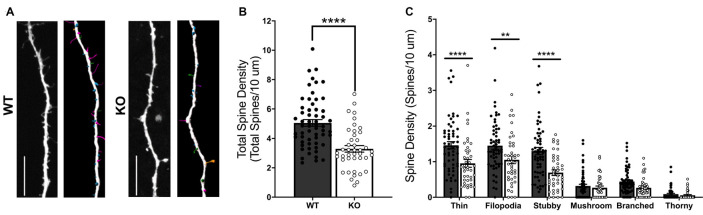
Dendritic spine analysis of WT and *Fmr1* knockout (KO) striatal MSNs at day *in vitro* (DIV) 14 (**A**; scale bars 10 μm). KO cells have a significant deficit in total dendritic spine density (**B**; unpaired *t*-test), as well as deficits in thin, filopodia, and stubby spine type densities (**C**; two-way RM ANOVA). Significant Bonferroni comparisons are indicated (**p < 0.01, ****p < 0.0001); data shown are mean ± SEM.

To more clearly determine the role of FMRP in regulating excitatory postsynaptic structure, we next compared dendritic spine density and morphology in cultured WT and *Fmr1* KO MSNs transfected with mCherry plasmid at DIV 14 (Figure 2A). An unpaired *t*-test showed a significant difference in total spine density between WT and KO cells (*t*_(98)_ = 5.382, *p* < 0.0001; Figure 2B), with *Fmr1* KO cells having significantly lower overall density. All spines were classified based on structural features (see “Materials and Methods” section) as thin, filopodia, stubby, mushroom, branched, or thorny type. Two-way mixed ANOVA indicated a significant interaction between genotype and spine classification (*F*_(5,490)_ = 5.323, *p* < 0.0001). Follow-up analysis revealed significant SMEs of genotype for thin (*p* < 0.0001), filopodia (*p* < 0.01), and stubby (*p* < 0.0001) spine types (Figure 2C), with the KO group having significantly lower spine density than the WT for each.

### FMRP’s RNA-Binding Domains Mediate Striatal Synaptic Markers

**Figure 3:**
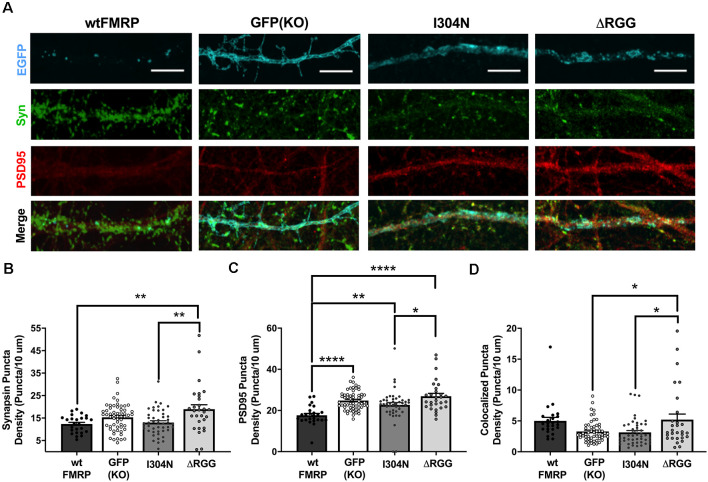
Expression of presynaptic (synapsin I; green) and postsynaptic (PSD95; red) markers at day *in vitro* (DIV) 14 in *Fmr1* knockout (KO) MSNs transfected with either green fluorescent protein (GFP) alone, or various forms of EGFP-tagged FMRP (**A**; scale bars are 10 μm). Cells transfected with ΔRGG-FMRP have a higher density of synapsin than those transfected with wt- or I304N-FMRP (**B**; one-way ANOVA). Cells transfected with wt-FMRP had significantly decreased density of PSD95 puncta, while those transfected with I304N- or ΔRGG-FMRP did not differ from GFP (KO) controls (**C**; one-way ANOVA). Cells transfected with ΔRGG-FMRP had a greater density of colocalized synapsin and PSD95 puncta compared to those transfected with I304N-FMRP and GFP (KO) controls (**D**; one-way ANOVA). Significant Bonferroni comparisons are indicated (*p < 0.05, **p < 0.01, ****p < 0.0001); data shown are mean ± SEM.

Acute expression of FMRP drives synapse elimination in hippocampal cells in a manner dependent on its KH2 RNA-binding domain (Pfeiffer and Huber, 2007). Given our basal findings, we next wanted to determine the effect of acute expression of FMRP, as well as the role of its KH2 and RGG RNA-binding domains, in regulating striatal excitatory synapse number. To do so, we compared pre-, post-, and colocalized synaptic puncta density in *Fmr1* KO cells transfected with plasmids expressing wtFMRP-EGFP, GFP alone, I304N-FMRP-EGFP, or ΔRGG-FMRP-EGFP at the DIV 14 time point (Figure 3A). For density of synapsin I puncta, one-way ANOVA showed a significant main effect of plasmid (*F*_(3,152)_ = 5.67, *p* = 0.001), with Bonferroni *post hoc* analysis indicating significantly greater density in the ΔRGG group compared to either the wtFMRP (*p* < 0.01) or I304N (*p* < 0.01) groups (Figure 3B). Analysis of PSD95 puncta density also revealed a significant main effect of plasmid (*F*_(3,152)_ = 11.5, *p* < 0.0001). Follow-up testing showed that cells receiving the wtFMRP plasmid had significantly lower PSD95 density compared to all other groups (GFP, *p* < 0.0001; I304N, *p* < 0.01; ΔRGG, *p* < 0.0001), and PSD95 density for the I304N group was significantly lower than that of the ΔRGG group (*p* < 0.05; Figure 3C). One-way ANOVA of colocalized puncta densities showed a significant main effect of plasmid (*F*_(3,152)_ = 5.262, *p* < 0.01), with Bonferroni follow-up analysis indicating that puncta density in the ΔRGG group was significantly higher than that of the GFP (KO; *p* < 0.05) and I304N (*p* < 0.05) groups (Figure 3D).

### FMRP’s RNA-Binding Domains Mediate MSN Dendritic Spine Morphology

**Figure 4:**
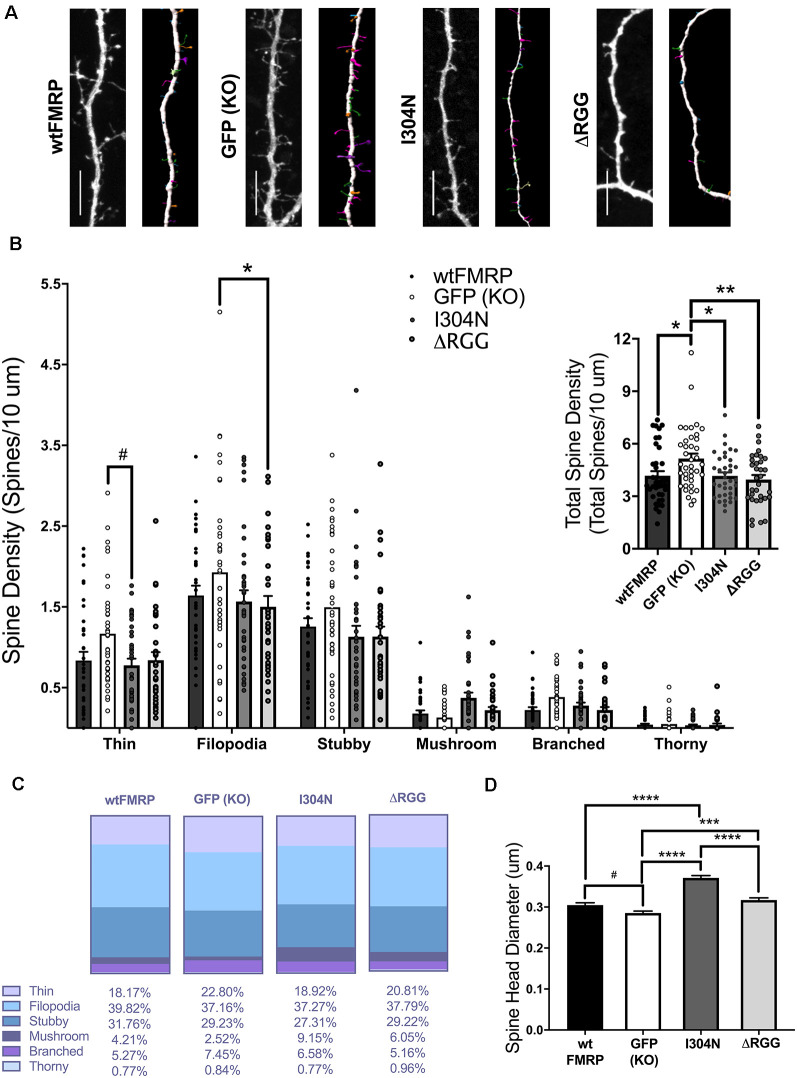
Dendritic spine analysis of striatal MSNs at day *in vitro* (DIV) 14, following transfection with plasmids expressing mCherry and either wtFMRP-EGFP, GFP, I304N-FMRP-EGFP, or ΔRGG-FMRP-EGFP (**A**; scale bars 10 μm). Groups receiving either wtFMRP-or mutant FMRP-expressing plasmids had significantly decreased total spine density compared to the GFP (*Fmr1* KO) control group (**B**; one-way ANOVA). The ΔRGG group had a significantly decreased density of filopodia spines, and the I304N group trended towards significantly decreased thin spines, compared to GFP (KO) control (**B**; two-way RM ANOVA). Relative distribution of spine types for each group is shown in panel **(C)**. Compared to the GFP (KO) group, spines in the I304N and ΔRGG group had greater spine head diameter (**D**; one-way ANOVA). Bonferroni comparisons are indicated (^#^*p* < 0.1, *p < 0.05, **p < 0.01, ***p < 0.001, ****p < 0.0001); data shown are mean ± SEM.

Given our findings that FMRP’s RNA-binding domains are involved in regulating synapse, and particularly PSD95, density, we next compared dendritic spine density and morphology between *Fmr1* KO cells transfected with plasmids expressing mCherry (for spine analysis) and either wtFMRP-EGFP, GFP, I304N-FMRP-EGFP, or ΔRGG-FMRP-EGFP (Figure 4A). For total spine density, one-way ANOVA revealed a significant main effect of plasmid (*F*_(3,143)_ = 4.586, *p* < 0.01), with Bonferroni *post hoc* analyses indicating significant decreases in spine density in the wtFMRP (*p* < 0.05), I304N (*p* < 0.05), and ΔRGG (*p* < 0.01) groups compared to the GFP (KO) control group (Figure 4B, inset). When spines were considered by type, a two-way mixed ANOVA showed a significant interaction between spine classification and plasmid (*F*_(15,715)_ = 1.76, *p* < 0.05). Follow-up Bonferroni analysis indicated that expression of ΔRGG significantly decreased the density of filopodia spines (*p* < 0.05), and the I304N group trended towards a significantly decreased thin spine density (*p* = 0.067), each compared to the GFP (KO) group (Figure 4B). The relative frequency distribution of spine types for each group is displayed in Figure 4C. We also observed differences in spine head diameter between groups, with one-way ANOVA showing a significant main effect of plasmid (*F*_(3,5241)_ = 53.08, *p* < 0.0001). Bonferroni *post hoc* analyses indicated that, compared to the GFP (KO) group, spines in the I304N and ΔRGG groups had significantly greater average head diameter (*p* < 0.0001 and *p* < 0.001, respectively), and there was a trend toward significance for wtFMRP spines to be of greater head diameter than GFP (KO; *p* = 0.055). In addition, the I304N group spines had significantly greater average head diameter than either the wtFMRP (*p* < 0.0001) or the ΔRGG (*p* < 0.0001) groups (Figure 4D).

## Discussion

Much of what is known about the neural function of FMRP, an RNA-binding protein that regulates RNA transport, stability, and translation, has been discovered in cortex and hippocampus (Huber et al., 2002; Antar et al., 2006; Grossman et al., 2006; Zang et al., 2013). Work done in hippocampal cells suggests key roles for FMRP and its KH2 RNA-binding domain in reducing synapse number (Pfeiffer and Huber, 2007). Using a cortical-striatal co-culture system, here we report findings consistent with a basal role for FMRP in promoting striatal dendritic spine and excitatory synapse number. Striatal WT MSNs show a significant increase in synapse count, associated with an increase in postsynaptic marker (PSD95) expression, which emerges between DIV 10 and 14. During the same time frame, *Fmr1* KO MSNs fail to increase PSD95 expression and at DIV 14 show an overall deficit in spine density compared to WT MSNs. On the other hand, similar to hippocampal cells, acute expression of FMRP in KO co-culture reduces striatal MSN spine density at DIV 14, though colocalized puncta do not show the same effect. By also making comparisons with mutant forms of FMRP, we show complex and unexpected roles for FMRP’s RNA-binding domains in this process.

Measuring basal differences in *Fmr1* KO and WT MSNs in co-culture, we find a reduction in colocalized synapsin I and PSD95 puncta in KO cells at DIV 14 that is accompanied by deficits in thin, filopodia, and stubby spine densities. While thin and filopodia spine types are considered immature and generally represent newly formed spines that will be either eliminated or stabilized into more mature, sometimes larger spine morphologies (Maletic-Savatic et al., 1999; Holtmaat et al., 2005; Petrak et al., 2005; Zuo et al., 2005), stubby spines are considered more stable and likely to contribute to stronger synaptic connections (Kasai et al., 2003). Comparing *Fmr1* KO to WT MSNs in slices prepared from NAc shell, we previously helped to identify a basal deficit in stubby spines, in particular, in adult KO mice (Smith et al., 2014). Our current *in vitro* findings strengthen our *in vivo* observations and suggest that these striatal deficits in spine morphology in FXS may be present across the lifespan. Observations in early postnatal hippocampal development (first week) similarly suggest that postsynaptic expression of FMRP promotes synapse function and maturation (Zang et al., 2013), and by adulthood, hippocampal, as well as cortical, *Fmr1* KO spine deficits generally manifest as excessive numbers of immature spine types (Antar et al., 2006; Grossman et al., 2006; He and Portera-Cailliau, 2013). Work performed in cultured *Fmr1* KO cortical cells identifies involvement of the synaptic adhesion molecule calsyntenin-1 (CLSTN1), an FMRP target mRNA (Darnell et al., 2011), as a potential mechanism for FMRP-mediated spine stabilization (Cheng et al., 2019). Indeed, it may be that a role for FMRP in spine stabilization is at the heart of these different adult morphological observations, manifesting differently depending on regional or cell type environmental conditions, including those that drive FMRP-dependent synapse elimination (Pfeiffer et al., 2010) to a greater or lesser degree. Notably, we and others have reported increased MSN spine densities, of either elongated (>1 μm; Neuhofer et al., 2015) or thin type (Smith et al., 2014), more similar to hippocampus or cortex, for the NAc core subregion of *Fmr1* KO mice, indicating that absence of FMRP *in vivo* drives different dendritic phenotypes even within striatal subregions.

In contrast to observations under basal conditions, we find that acute expression of wtFMRP-GFP in striatal *Fmr1* KO cells significantly *decreases* both PSD95 puncta number and overall dendritic spine density compared to GFP expression alone (KO). Indeed, FMRP is known to mediate activity-dependent synapse weakening and elimination in the brain (Weiler and Greenough, 1999; Pfeiffer et al., 2010; Zang et al., 2013). For example, acute expression of wtFMRP in *Fmr1* KO hippocampal dissociated and slice cultures reduces total PSD95 and synapsin (unspecified) puncta, an interpretation that was bolstered by measurements of miniature excitatory postsynaptic current frequency (Pfeiffer and Huber, 2007). However, in our study, while we also observe reductions in PSD95 puncta counts and spine density in wtFMRP-treated cells, a similar effect is not present for colocalized puncta—a feature representative of functional synapses (Ippolito and Eroglu, 2010; Verstraelen et al., 2018). In fact, while numbers of colocalized puncta align well with overall spine density in our studies of basal conditions, when we perturb the KO cell environment with acute availability of FMRP, these outcomes no longer align, at least at the examined time point (DIV 14). For example, I304N- and ΔRGG-FMRP expression each result in overall spine densities that are significantly below KO and comparable to wtFMRP; however, despite this fact, ΔRGG-FMRP-treated striatal cells have normal levels of colocalized puncta, expressing significantly more than either the GFP (KO) or I304N-FMRP groups. It may be that greater pre- and postsynaptic availability increases opportunities for successful colocalization in the ΔRGG group; however, future studies should determine whether such putative synaptic alignments are occurring with proper relevance to experience and whether they affect behavioral outcomes *in vivo*.

Notably, while both mutant forms of FMRP decrease overall spine density, they fail to decrease PSD95 puncta. These seemingly contradictory outcomes could occur if existing spines in these groups express greater numbers of postsynaptic (PSD95) proteins, or “nanomodules,” a phenomenon recently described (Hruska et al., 2018). While the current study does not address this question, we do observe that I304N- and ΔRGG-FMRP-expressing cells exhibit specific decreases in immature spine types (thin and filopodia, respectively), whereas wtFMRP-expressing cells show a more general decrease in density across multiple types. Spines in the I304N group also, on average, show a significantly greater average spine head diameter than that of any other group. Spine head diameter and volume positively correlate with PSD95 expression, and not all new or transient spines (i.e., thin, filopodia types) will acquire PSD95 puncta (Cane et al., 2014), so it may be that FMRP’s KH2 and RGG domains are not required for elimination of immature dendritic spines that lack stable incorporation of PSD95. We emphasize that the RGG domain is dispensable for synapse elimination in hippocampal cells, while the 1304N mutation has been shown to disrupt this and other hippocampal cellular functions (Pfeiffer and Huber, 2007). The fact that our results in striatal cells do not entirely align with synapse elimination after acute presentation of FMRP suggests that we may be observing mixed states of synapse and spine elimination, stabilization, and/or homeostatic recovery. These outcomes are likely complicated by the myriad roles FMRP plays in structural plasticity, dependent in different ways on the protein domains examined here. As one example, FMRP’s RGG domain is required to limit forward trafficking of N-type Ca^2+^ channels to the presynaptic active zone (Ferron et al., 2020), a process important to early synaptogenesis (Pravettoni et al., 2000; Rieckhof et al., 2003), which may contribute to the increased synapsin labeling and normal levels of colocalized puncta that we observe in the ΔRGG-FMRP-expressing group. In any case, our findings add to our understanding of FXS, suggesting distinctions in FMRP’s role for different brain regions and/or cell types.

We note that striatal cells in experiments described here were co-cultured alongside cortical cells of the same genotype. In transfection experiments, while only MSNs expressing the transfected plasmid (GFP+) were analyzed, these cells likely received excitatory cortical and inhibitory MSN collateral input from other successfully transfected cells, as well as input from *Fmr1* KO untransfected cells. Thus, we cannot rule out the influence of abnormal presynaptic signaling, such as that described previously (Deng et al., 2013; Patel et al., 2013). However, a study specifically investigating cortico-striatal signaling reported enhanced GABAergic, but normal glutamatergic, transmission onto striatal cells in *Fmr1* KO mice (Centonze et al., 2008), suggesting that our findings do not likely result from abnormal excitatory cortical cell input. In any case, there is abundant evidence of FMRP’s importance in the postsynapse across various brain regions in many aspects of synapse plasticity, including synaptogenesis (Wang et al., 2018), synaptic scaling and synaptic strength (Soden and Chen, 2010), as well as synapse elimination (Pfeiffer and Huber, 2007). We also note that general KO striatal spine deficits were the impetus for this work. While many spines on MSNs appose cortical projections, axonal collateral terminals from other nearby MSNs, despite being inhibitory, are also commonly found contributing to asymmetric synapses in the striatum (Wilson and Groves, 1980), thus we did not differentiate presynaptic puncta by type. Lastly, because wtFMRP is introduced to *Fmr1* KO cells in our acute transfection studies, it is possible that the previous absence of FMRP underlies the discrepancy between these and our basal condition experiments. Indeed, FMRP is normally present during embryonic development and its absence results in, among other things, aberrant gene expression and impaired differentiation of neural progenitor cells (Sunamura et al., 2018). Additional studies will be needed to parse the influence of FMRP’s pre- and postsynaptic functions, as well as potential developmental roles, on striatal MSN dendritic morphology.

FMRP is a critical player in synapse regulation, with much of its function discovered in brain regions that are characterized by relatively high densities of glutamatergic neurons and excitatory transmission. The striatum, largely made up of relatively quiescent, GABAergic inhibitory MSNs, plays a major role in motor activation, as well as social and repetitive behaviors, all of which are detrimentally affected in FXS. While FMRP-mediated synapse elimination is likely contributing to observed outcomes in striatal MSNs, similar to findings in hippocampal cells, results from both our basal and acute transfection studies indicate a critical role for FMRP in striatal synapse stabilization during this early time period. Given that forms of FXS and intellectual disability have been linked to mutations disrupting specific domains of FMRP, including KH2 and RGG, our work also supports the idea that appropriately nuanced treatment approaches may be most effective. Moving forward, it will be important to investigate FMRP’s pre- and postsynaptic functions in striatum and the consequences of both total and domain-specific disruption of FMRP on both cell physiological and behavioral function.

## Data Availability Statement

The raw data supporting the conclusions of this article will be made available by the authors, without undue reservation.

## Ethics Statement

The animal study was reviewed and approved by the Texas A&M University Institutional Care and Use Committee.

## Author Contributions

JH contributed to the design, acquisition, analysis, interpretation, and writing of the manuscript. KC contributed to the acquisition, analysis, and revision of the manuscript. YG contributed to the design, acquisition, and revision of the manuscript. LS contributed to the design, acquisition, analysis, interpretation, and writing of the manuscript.

## Conflict of Interest

The authors declare that the research was conducted in the absence of any commercial or financial relationships that could be construed as a potential conflict of interest.
