# Supplementary material for: The Fragile X Mental Retardation Protein Regulates Striatal Medium Spiny Neuron Synapse Density and Dendritic Spine Morphology
Source: Front Mol Neurosci. 2020 Sep 10;13:161. doi: 10.3389/fnmol.2020.00161 (PMC7511717; doi:10.3389/fnmol.2020.00161)
Supplement: Supplementary file 1 [file Table_1.pdf]

Table S1.

**Figure**	**Statistical Analysis**	**Dependent Variable**	**Factor(s)**	**DF**	**F / t / X^2 value**	**P value**
1B	Two-Way ANOVA	Synapsin WT vs KO, 10 DIV and 14 DIV	day	1, 236	0.2973	0.05861
			genotype	1, 236	2.362	0.1257
			day x genotype	1, 236	0.0004437	0.09832
1C	Two-Way ANOVA	PSD95 WT vs KO, 10 DIV and 14 DIV	day	1, 236	17.09	0.00004954
			genotype	1, 236	0.4857	0.4865
			**day x genotype**	**1, 236**	**4.134**	**0.04314**
	Bonferroni Pairwise Comparision	PSD95, 10 DIV vs 14 DIV (WT only)	**day**			**0.0001235**
	Bonferroni Pairwise Comparision	PSD95, 10 DIV vs 14 DIV (KO only)	day			0.8245
	Bonferroni Pairwise Comparision	PSD95, WT vs KO (10 DIV)	genotype			> 0.9999
	Bonferroni Pairwise Comparision	PSD95, WT vs KO (14 DIV)	genotype			0.2576
1D	Two-Way ANOVA	Colocalized Puncta, WT vs KO, 10 DIV and 14 DIV	**day**	**1, 236**	**13.13**	**0.0004**
			**genotype**	**1, 236**	**6.639**	**0.0106**
			day x genotype	1, 236	1.889	0.1706

**Figure**	**Statistical Analysis**	**Dependent Variable**	**Factor(s)**	**DF**	**F / t / X^2 value**	**P value**
2B	Unpaired *t* Test	Total Dendritic Spine Density, WT vs KO	**genotype**	**98**	**5.382**	**0.0000005044**
2C	Two-Way RM ANOVA	Dendritic Spine Density by Type, WT vs KO	spine type	5, 490	95.3	< 0.0000000001
			genotype	1, 98	28.96	0.0000005
			**spine type x genotype**	**5, 490**	**5.323**	**0.00008944**
	Bonferroni Pairwise Comparision	WT vs KO, Thin Spine Density	**genotype**			**0.00003318**
	Bonferroni Pairwise Comparision	WT vs KO, Filopodia Spine Density	**genotype**			**0.002223**
	Bonferroni Pairwise Comparision	WT vs KO, Stubby Spine Density	**genotype**			**0.0000004**
	Bonferroni Pairwise Comparision	WT vs KO, Mushroom Spine Density	genotype			> 0.9999
	Bonferroni Pairwise Comparision	WT vs KO, Branched Spine Density	genotype			0.6694
	Bonferroni Pairwise Comparision	WT vs KO, Thorny Spine Density	genotype			> 0.9999

**Figure**	**Statistical Analysis**	**Dependent Variable**	**Factor(s)**	**DF**	**F / t / X^2 value**	**P value**
3B	One-Way ANOVA	Synapsin WT vs GFP vs I304N vs ΔRGG	**plasmid**	**3, 152**	**5.668**	**0.0010**
	Bonferroni Pairwise Comparision	Synapsin WT vs GFP	plasmid			0.4099
	Bonferroni Pairwise Comparision	Synapsin WT vs I304N	plasmid			> 0.9999
	Bonferroni Pairwise Comparision	Synapsin WT vs ΔRGG	**plasmid**			**0.0032**
	Bonferroni Pairwise Comparision	Synapsin GFP vs I304N	plasmid			0.6008
	Bonferroni Pairwise Comparision	Synapsin GFP vs ΔRGG	plasmid			0.1368
	Bonferroni Pairwise Comparision	Synapsin I304N vs ΔRGG	**plasmid**			**0.0027**
3C	One-Way ANOVA	PSD95 WT vs GFP vs I304N vs ΔRGG	**plasmid**	**3, 152**	**11.5**	**0.00000078**
	Bonferroni Pairwise Comparision	PSD95 WT vs GFP	**plasmid**			**0.00002**
	Bonferroni Pairwise Comparision	PSD95 WT vs I304N	**plasmid**			**0.0076**
	Bonferroni Pairwise Comparision	PSD95 WT vs ΔRGG	**plasmid**			**0.00000099**
	Bonferroni Pairwise Comparision	PSD95 GFP vs I304N	plasmid			0.546
	Bonferroni Pairwise Comparision	PSD95 GFP vs ΔRGG	plasmid			0.826
	Bonferroni Pairwise Comparision	PSD95 I304N vs ΔRGG	**plasmid**			**0.029**
3D	One-Way ANOVA	Colocalized Puncta, WT vs GFP vs I304N vs ΔRGG	**plasmid**	**3, 152**	**5.262**	**0.0018**
	Bonferroni Pairwise Comparision	Colocalized Puncta WT vs GFP	plasmid			0.0735
	Bonferroni Pairwise Comparision	Colocalized Puncta WT vs I304N	plasmid			0.0593
	Bonferroni Pairwise Comparision	Colocalized Puncta WT vs ΔRGG	plasmid			> 0.9999
	Bonferroni Pairwise Comparision	Colocalized Puncta GFP vs I304N	plasmid			> 0.9999
	Bonferroni Pairwise Comparision	Colocalized Puncta GFP vs ΔRGG	**plasmid**			**0.0200**
	Bonferroni Pairwise Comparision	Colocalized Puncta I304N vs ΔRGG	**plasmid**			**0.0167**

**Figure**	**Statistical Analysis**	**Dependent Variable**	**Factor(s)**	**DF**	**F / t / X^2 value**	**P value**
4B	One-Way ANOVA	Total Dendritic Spine Density, WT vs GFP vs I304N vs ΔRGG	**plasmid**	**3, 143**	**4.586**	**0.0043**
	Bonferroni Pairwise Comparision	Total Spine Density, WT vs GFP	**plasmid**			**0.037**
	Bonferroni Pairwise Comparision	Total Spine Density, WT vs I304N	plasmid			> 0.9999
	Bonferroni Pairwise Comparision	Total Spine Density, WT vs ΔRGG	plasmid			> 0.9999
	Bonferroni Pairwise Comparision	Total Spine Density, GFP vs I304N	**plasmid**			**0.0333**
	Bonferroni Pairwise Comparision	Total Spine Density, GFP vs ΔRGG	**plasmid**			**0.0077**
	Bonferroni Pairwise Comparision	Total Spine Density, I304N vs ΔRGG	plasmid			> 0.9999
	Two-Way RM ANOVA	Dendritic Spine Density by Type, WT vs GFP vs I304N vs ΔRGG	spine type	5, 715	220.3	< 0.000000000000001
			plasmid	3, 143	4.586	0.00426
			**spine type x plasmid**	**15, 715**	**1.76**	**0.036**
	Bonferroni Pairwise Comparision	Thin Spine Density, WT vs GFP	plasmid			0.277
	Bonferroni Pairwise Comparision	Thin Spine Density, WT vs I304N	plasmid			> 0.9999
	Bonferroni Pairwise Comparision	Thin Spine Density, WT vs ΔRGG	plasmid			> 0.9999
	Bonferroni Pairwise Comparision	Thin Spine Density, GFP vs I304N	plasmid			0.067
	Bonferroni Pairwise Comparision	Thin Spine Density, GFP vs ΔRGG	plasmid			0.409
	Bonferroni Pairwise Comparision	Thin Spine Density, I304N vs ΔRGG	plasmid			> 0.9999
	Bonferroni Pairwise Comparision	Filopodia Spine Density, WT vs GFP	plasmid			0.7759
	Bonferroni Pairwise Comparision	Filopodia Spine Density, WT vs I304N	plasmid			> 0.9999
	Bonferroni Pairwise Comparision	Filopodia Spine Density, WT vs ΔRGG	plasmid			> 0.9999
	Bonferroni Pairwise Comparision	Filopodia Spine Density, GFP vs I304N	plasmid			0.1431
	Bonferroni Pairwise Comparision	Filopodia Spine Density, GFP vs ΔRGG	**plasmid**			**0.0362**
	Bonferroni Pairwise Comparision	Filopodia Spine Density, I304N vs ΔRGG	plasmid			> 0.9999
	Bonferroni Pairwise Comparision	Stubby Spine Density, WT vs GFP	plasmid			> 0.9999
	Bonferroni Pairwise Comparision	Stubby Spine Density, WT vs I304N	plasmid			> 0.9999
	Bonferroni Pairwise Comparision	Stubby Spine Density, WT vs ΔRGG	plasmid			> 0.9999
	Bonferroni Pairwise Comparision	Stubby Spine Density, GFP vs I304N	plasmid			0.133
	Bonferroni Pairwise Comparision	Stubby Spine Density, GFP vs ΔRGG	plasmid			0.1825
	Bonferroni Pairwise Comparision	Stubby Spine Density, I304N vs ΔRGG	plasmid			> 0.9999
	Bonferroni Pairwise Comparision	Mushroom Spine Density, WT vs GFP	plasmid			> 0.9999
	Bonferroni Pairwise Comparision	Mushroom Spine Density, WT vs I304N	plasmid			> 0.9999
	Bonferroni Pairwise Comparision	Mushroom Spine Density, WT vs ΔRGG	plasmid			> 0.9999
	Bonferroni Pairwise Comparision	Mushroom Spine Density, GFP vs I304N	plasmid			> 0.9999
	Bonferroni Pairwise Comparision	Mushroom Spine Density, GFP vs ΔRGG	plasmid			> 0.9999
	Bonferroni Pairwise Comparision	Mushroom Spine Density, I304N vs ΔRGG	plasmid			> 0.9999
	Bonferroni Pairwise Comparision	Branched Spine Density, WT vs GFP	plasmid			> 0.9999
	Bonferroni Pairwise Comparision	Branched Spine Density, WT vs I304N	plasmid			> 0.9999
	Bonferroni Pairwise Comparision	Branched Spine Density, WT vs ΔRGG	plasmid			> 0.9999
	Bonferroni Pairwise Comparision	Branched Spine Density, GFP vs I304N	plasmid			> 0.9999
	Bonferroni Pairwise Comparision	Branched Spine Density, GFP vs ΔRGG	plasmid			> 0.9999
	Bonferroni Pairwise Comparision	Branched Spine Density, I304N vs ΔRGG	plasmid			> 0.9999
	Bonferroni Pairwise Comparision	Thorny Spine Density, WT vs GFP	plasmid			> 0.9999
	Bonferroni Pairwise Comparision	Thorny Spine Density, WT vs I304N	plasmid			> 0.9999
	Bonferroni Pairwise Comparision	Thorny Spine Density, WT vs ΔRGG	plasmid			> 0.9999
	Bonferroni Pairwise Comparision	Thorny Spine Density, GFP vs I304N	plasmid			> 0.9999
	Bonferroni Pairwise Comparision	Thorny Spine Density, GFP vs ΔRGG	plasmid			> 0.9999
	Bonferroni Pairwise Comparision	Thorny Spine Density, I304N vs ΔRGG	plasmid			> 0.9999
4D	One-Way ANOVA	Spine Head Maximum Diameter, WT vs GFP vs I304N vs ΔRGG	**plasmid**	**3, 5241**	**53.08**	**< 0.000000000000001**
	Bonferroni Pairwise Comparision	Spine Head Maximum Diameter, WT vs GFP	plasmid			0.055
	Bonferroni Pairwise Comparision	Spine Head Maximum Diameter, WT vs I304N	**plasmid**			**< 0.000000000000001**
	Bonferroni Pairwise Comparision	Spine Head Maximum Diameter, WT vs ΔRGG	plasmid			0.7554
	Bonferroni Pairwise Comparision	Spine Head Maximum Diameter, GFP vs I304N	**plasmid**			**< 0.000000000000001**
	Bonferroni Pairwise Comparision	Spine Head Maximum Diameter, GFP vs ΔRGG	**plasmid**			**< 0.000186**
	Bonferroni Pairwise Comparision	Spine Head Maximum Diameter, I304N vs ΔRGG	**plasmid**			**< 0.0000000000207**
